# Supplementary figures and images for: Modeling of axonal endoplasmic reticulum network by spastic paraplegia proteins
Source: eLife. 2017 Jul 25;6:e23882. doi: 10.7554/eLife.23882 (PMC5576921; doi:10.7554/eLife.23882)

*Wild type*

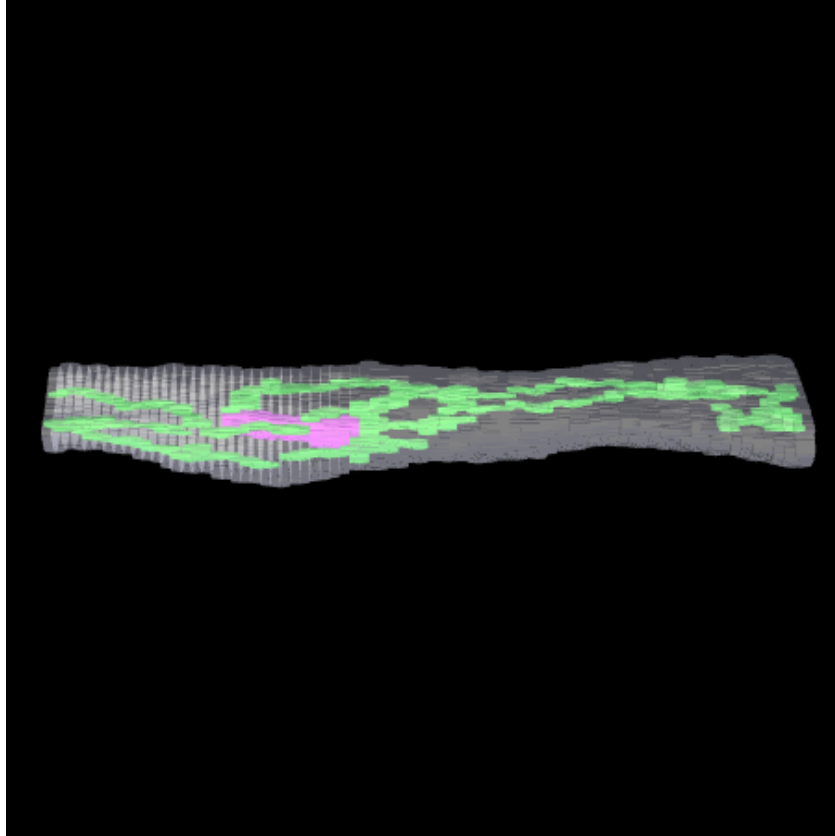

Supplement: Supplementary file 2. — Reconstruction is generated from 75 serial 60 nm sections, and shows ER (green), a mitochondrion (magenta), and plasma membrane (gray). DOI: http://dx.doi.org/10.7554/eLife.23882.025 [file elife-23882-supp2.pdf]

*RtnI1<sup>-</sup> ReepA<sup>-</sup> ReepB<sup>-</sup>*

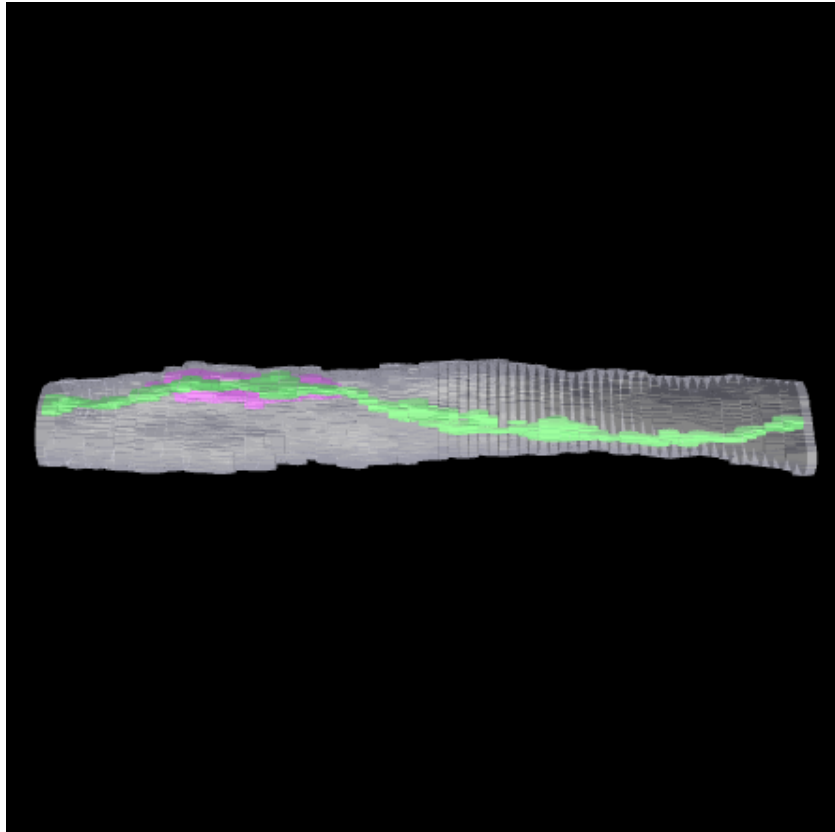

Supplement: Supplementary file 3. — Reconstruction is generated from 75 serial 60 nm sections, and shows ER (green), a mitochondrion (magenta), and plasma membrane (gray). DOI: http://dx.doi.org/10.7554/eLife.23882.026 [file elife-23882-supp3.pdf]

*RtnI1<sup>-</sup> ReepA<sup>-</sup> ReepB<sup>-</sup>*

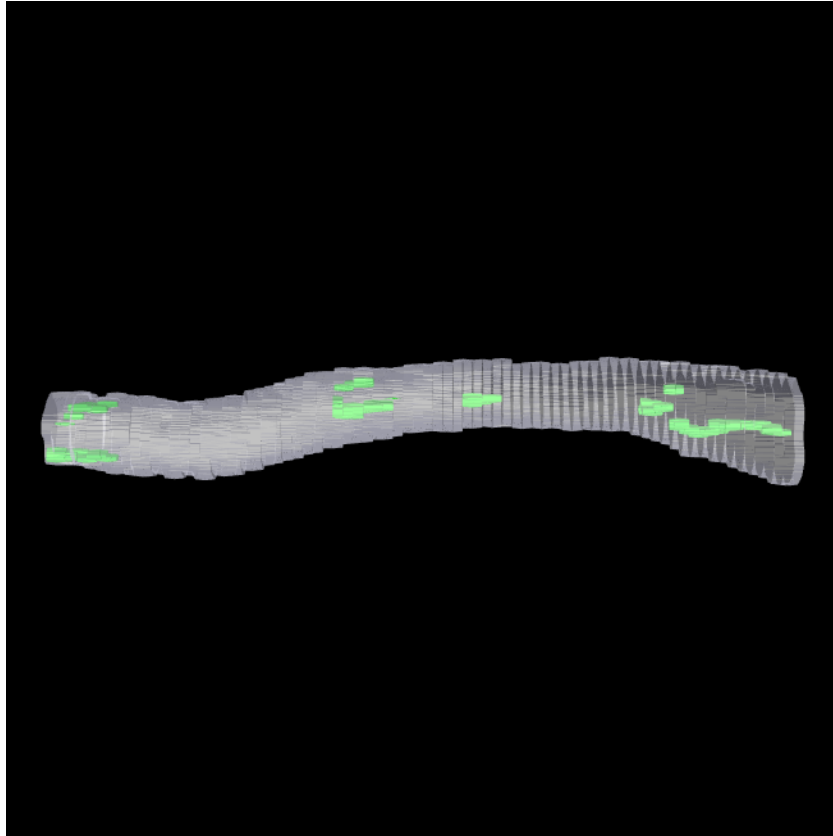

Supplement: Supplementary file 4. — The reconstruction was generated from 75 serial sections of 60 nm each. ER is in green and the plasma membrane in gray. DOI: http://dx.doi.org/10.7554/eLife.23882.027 [file elife-23882-supp4.pdf]
